# Clinicopathological Significance, Related Molecular Changes and Tumor Immune Response Analysis of the Abnormal SWI/SNF Complex Subunit PBRM1 in Gastric Adenocarcinoma

**DOI:** 10.3389/pore.2022.1610479

**Published:** 2022-07-19

**Authors:** Zhiyi Zhou, Dandan Huang, Shudong Yang, Jiabei Liang, Xuan Wang, Qiu Rao

**Affiliations:** ^1^ Department of Pathology, The Affiliated Wuxi People’s Hospital of Nanjing Medical University, Wuxi, China; ^2^ Digestive Endoscopic Center, The Affiliated Wuxi People’s Hospital of Nanjing Medical University, Wuxi, China; ^3^ Department of Pathology, Jinling Hospital, Nanjing University School of Medicine, Nanjing, China

**Keywords:** prognosis, PD-L1, gastric adenocarcinoma (GAC), PBRM1, microsatellite stability, tumor-infiltrating lymphocytes (TIL)

## Abstract

**Background:** PBRM1 gene abnormalities were recently found to play a role in tumor development and tumor immune activity. This article will explore the clinicopathological and molecular changes and tumor immune activity of the abnormal SWI/SNF complex subunit PBRM1 in gastric adenocarcinoma (GAC) and its significance.

**Methods:** The cBioPortal, LinkedOmics and TISIDB datasets were used to analyze the abnormality of the PBRM1 gene in GAC and its relationship with prognosis, related molecular changes and tumor-infiltrating lymphocytes (TILs). In addition, 198 GAC cases were collected to further study the relationship between the loss/attenuation of PBRM1 expression and clinicopathology, prognosis, microsatellite stability, PD-L1 expression and TIL in GAC. DNA whole-exome sequencing was performed on 7 cases of gastric cancer with loss of PBRM1 expression.

**Results:** The cBioPortal data showed that PBRM1 deletion/mutation accounted for 7.32% of GAC and was significantly associated with several molecular changes, such as molecular subtypes of GAC. The LinkedOmics dataset showed that PBRM1 mutation and its promoter DNA methylation showed lower PBRM1 mRNA expression, and PBRM1 mutation cases showed significantly higher mRNA expression of PD-L1 (CD274). TISIDB data showed that PBRM1 abnormalities were significantly positively associated with multiple TILs. In our group of 198 cases, the loss/attenuation of PBRM1 expression was significantly positively correlated with intra-tumoral tumor infiltrating lymphocytes (iTILs) and deficient MMR and PD-L1 expression. Kaplan–Meier survival analysis showed that the overall survival of GAC patients with loss/attenuation of PBRM1 expression was significantly better (*p* = 0.023). iTIL was an independent prognostic factor of GAC. Loss of PBRM1 expression often co-occurs with mutations in other SWI/SNF complex subunit genes, and there are some repetitive KEGG signaling changes.

**Conclusion:** Abnormality of the PBRM1 gene may be related to the occurrence of some GACs and can affect tumor immune activity, thereby affecting clinicopathology and prognosis. It may be a potentially effective predictive marker for immunotherapy and a novel therapeutic approach associated with synthetic lethality.

## Introduction

Gastric adenocarcinoma (GAC) is a common malignant tumor, and patients with advanced GAC have a poor prognosis. At present, clinical trials of anti-PD-1 (programmed cell death protein-1)/PD-L1(Programmed Cell Death-Ligand 1) therapy for GAC have achieved good results. Microsatellite instability-high (MSI-H), high tumor mutational burden (TMB) or PD-L1 expression are the main indicators to predict its efficacy. However, the response to treatment remains highly variable, and some patients remain unresponsive to immunotherapy (1). It is therefore necessary to further study the related mechanisms of tumor immunity and to find specific and effective predictive biomarkers related to the tumor immune response.

Whole-exome sequencing revealed that genes encoding subunits of the SWItch/Sucrose Nonfermentable (SWI/SNF) complex are mutated in more than 20% of cancers, involving multiple cancer types (2). The SWI/SNF complex is a subfamily of adenosine triphosphate-dependent (ATP-dependent) chromatin remodeling proteins that regulate the access of transcription factors through control based on nucleosome topology remodeling and can recruit proteins to promote DNA repair through mechanisms such as nucleotide excision and double-strand break repair. Therefore, the loss or abnormality of its function may lead to genome instability and the occurrence and progression of tumors and can also enhance the antitumor immune response and affect the biological behavior of tumors (2). This complex includes the BRG1(BRM(Brahma)/SWI2 related gene 1)/BRM-associated factor complex (BRG1/BRM-associated factor, BAF) and polybromo-associated BAF complex (polybromo-associated BAF, PBAF). Polybromo 1(PBRM1) is an important accessory regulatory subunit of the latter. PBRM1 is localized to 3p21, and loss or inactivating mutations of PBRM1 can mediate the regulation of cell growth, migration, proliferation and differentiation in a variety of cancers and can be involved in tumor immune responses and related immune treatments (3). Therefore, we hypothesized that abnormal PBRM1 may play a role in the carcinogenesis and tumor immune activity of GAC.

This study sought to explore the relationship between PBRM1 gene abnormalities and the occurrence and development of GAC and tumor immune activity and to provide evidence for its potential use as a predictive marker for GAC immunotherapy. In this paper, cBioPortal, LinkedOmics and TISIDB datasets were used to analyze the abnormality of the PBRM1 gene in GAC and its relationship with prognosis, related molecular changes and tumor-infiltrating lymphocytes (TIL). GAC cases were collected to further verify and study the effect of PBRM1 loss/attenuation on clinicopathology and prognosis and analyze its relationship with microsatellite stability, PD-L1 expression and TILs in GAC.

## Materials and Methods

### Clinical Cases

Overall, 198 patients with advanced GAC who underwent radical gastrectomy from July 2015 to December 2016 in our department with follow-up information were retrospectively collected. Patient clinical information included age, sex, tumor site, histological differentiation, lymph node metastasis status, and TNM stage. This study was approved by the Ethics Committee of the Affiliated Wuxi People’s Hospital of Nanjing Medical University (No. KS202017). Overall survival (OS) was determined by telephone follow-up until April 2020. Clinical information of these cases was summarized in Supplementary Table S1.

## Methods

### TCGA and cBioPortal Database Analysis

The cBioPortal online platform (http://www.cbioportal.org/) was used to process TCGA-GC data (4). The GAC data (TCGA, Nature 2014) (Supplementary Table S2) were selected for analysis, and the PBRM1 deletion/mutation status of GAC and its related molecular changes were analyzed.

### LinkedOmics Dataset

The LinkedOmics (http://www.linkedomics.org/login.php) dataset contains multiomics data for 32 TCGA cancer types (5). LinkedOmics was used to analyze the correlation of PBRM1 mutation with PBRM1, PD-L1 (CD274) and CTLA4 mRNA expression and patient prognosis, as well as the correlation between PBRM1 mRNA expression and PBRM1 DNA methylation in GAC.

### TISIDB Analysis

The TISIDB database (http://cis.hku.hk/TISIDB) integrates 988 immune-related antitumor genes, high-throughput screening technology, molecular profiling, paracancer multiomics data and various immunological data from seven public databases (6). In this study, the TISIDB database was used to analyze the relationship between various TILs and PBRM1 gene expression, mutation, CNA (Copy Number Alteration) and gene methylation in GAC.

### Immunohistochemical Staining

Using the EnVision method, antibodies included PBRM1 (polyclonal antibody, A301-591A, 1:4000; Bethyl Laboratories), MLH1, MSH2, MSH6, PMS2 (clone numbers are ES05, FE11, EP49 and EP51, Dako company), and PD-L1 (mouse mAb 22C3, concentrated solution, 1:50 dilution, Dako company). Expression results were analyzed using a double-blind method. The primary antibody was replaced with PBS as a negative control.

According to the literature, normal epithelial cells, inflammatory cells and fibroblasts were used as internal positive controls; if the nuclear staining intensity was lost or significantly reduced (including the heterogeneous staining), it was classified as PBRM1 expression loss/attenuation (7). MSH2, MSH6, MLH1 and PMS2 are expressed in the nucleus, and the deletion of any protein is regarded as deficient DNA mismatch repair protein (dMMR), which is equivalent to MSI-H. The combined positive score (CPS) was used for PD-L1 interpretation, that is, the percentage of live positive tumor cells (membrane or cytoplasmic staining of any intensity) plus positive immune cells (lymphocytes, macrophages) divided by all live tumor cells, multiplied by 100. The result is expressed as a value from 0 to 100 (when it exceeds 100, it is counted as 100), and it is regarded as positive when ≥1.

### Evaluation of Stromal Tumor-Infiltrating Lymphocytes and Intra-Tumoral Tumor-Infiltrating Lymphocytes

Referring to the International Immuno-Oncology Biomarkers Working Group (IIOBWG) standards and related literature (8, 9,10), TIL refers to the infiltration of all mononuclear-like cells, including lymphocytes and plasma cells. Stromal TIL (sTIL) density was assessed using ×200 magnification and scored as the percentage of stromal area occupied by TILs to the total intratumoral stromal area. The patients were divided into two groups, “more” sTIL and “less” sTIL, with the median percentage as the cutoff. The presence of iTILs (intra-tumoral TILs) was defined as≥5 lymphocytes per high-power field.

### Whole Exome Sequencing

Genomic DNA from formalin-fixed paraffin-embedded (FFPE) samples was extracted using QIAamp DNA FFPE Tissue Kit (Qiagen)and fragmented by ultrasonication (Covaris). Libraries were prepared using the KAPA Hyper Prep Kit (KAPA Biosystems) (11), and the xGen Exome Research Panel (Integrated DNA Technologies) was used for exome capture. Enriched libraries were sequenced using an Illumina HiSeq 4000 platform with a mean coverage of ∼60X for the control and ∼150X for the tumor samples.

Paired-end sequencing data were aligned to the reference human genome hg19) using the Burrows-Wheeler Aligner (bwa-mem), and then were subjected to de-duplication, base quality recalibration and indel realignment using Picard (http://picard.sourceforge.net/) and the Genome Analysis Toolkit (GATK). Somatic single nucleotide variants (SNVs) were called by MuTect using paired normal controls, using the filtering criteria of >1% frequencies in the 1000 Genomes and dbSNP. Small insertions and deletions (indels) were detected using SCALPEL. Variants were further annotated by ANNOVAR. Gene-level copy ratios were calculated by CNVKit.

### Statistical Methods

A chi-square test was used to analyze the relationship between PBRM1 deletion/mutation (or loss/attenuation of PBRM1 expression) and various clinicopathological and molecular parameters. Survival analysis was performed using Kaplan–Meier to draw survival curves, and the log-rank test was used to compare differences in survival time. Cox regression was used to determine independent prognostic factors. SPSS 23.0 statistical software was used, and *p* < 0.05 was considered statistically significant.

## Results

### cBioPortal Data

**FIGURE 1:**
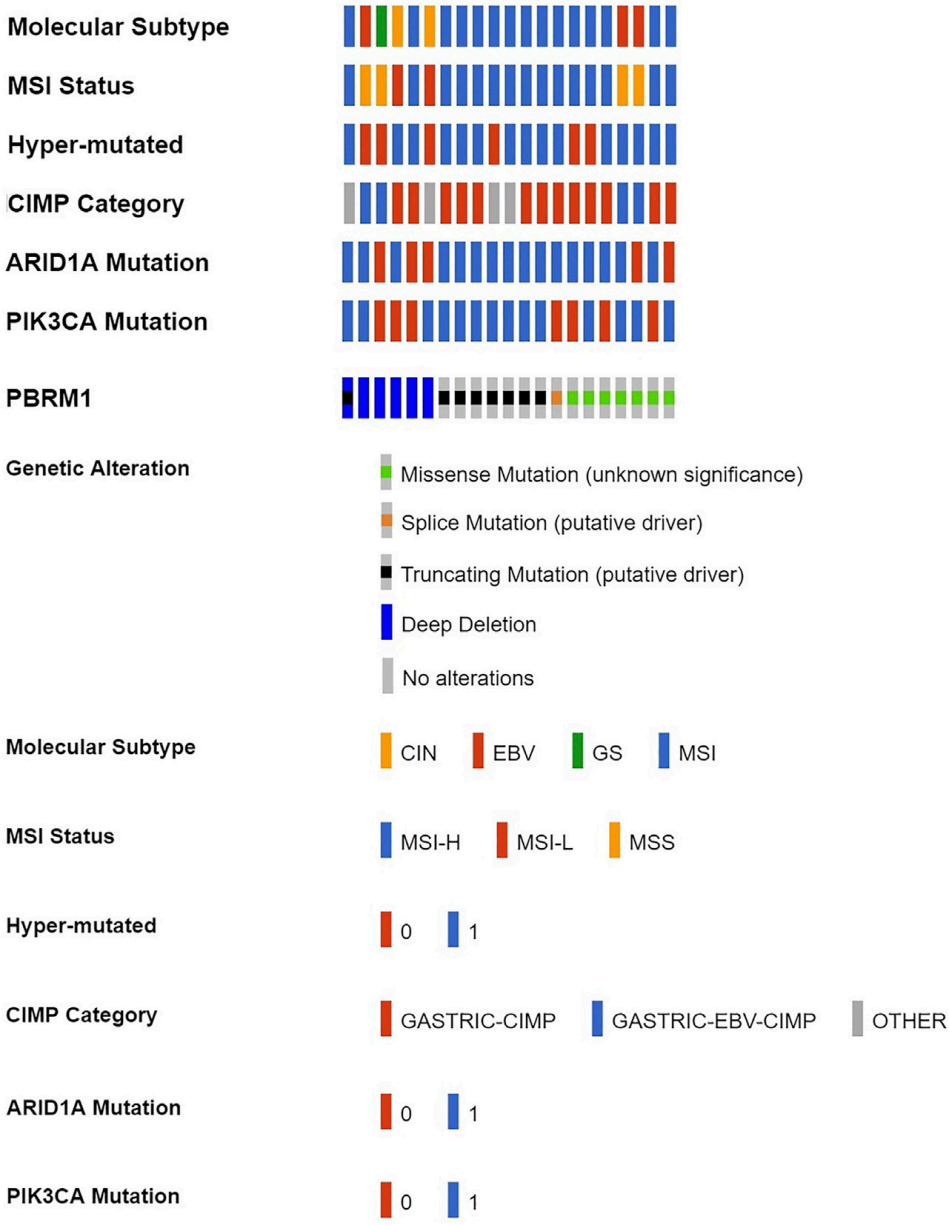
PBRM1 deletion/mutation in GAC and its relationship with various molecular alterations in cBioPortal data (CIN, chromosomal instability; EBV, Epstein-Barr virus type; GS, genome stability; MSI, microsatellite instability; MSH-H, microsatellite instability-High; MSH-L, microsatellite instability-Low; MSS, microsatellite stability; 0 = No, 1 = Yes).

According to cBioPortal data, 7.32% of GACs had PBRM1 deletions/mutations (Shown in Supplementary Table S3). PBRM1 deletion/mutation was significantly positively correlated with MSI status, hypermutation, ARID1A mutation, PIK3CA mutation and associated with molecular subtype and CpG island methylator phenotype (CIMP) class (Figure 1 and Table 1).

**TABLE 1:** Correlation of PBRM1 deletion/mutation with various molecular alterations in 287 TCGA GC cases.

Parameters	PBRM1 Deletion/mutation		χ^2^ value	*p* value
	Yes	No		
Molecular Subtype			35.993	0.000
EBV	3	22		
MSI	15	48		
GS	1	53		
CIN	2	143		
MSI Status			32.656	0.000
MSS	4	176		
MSI-L	2	42		
MSI-H	15	48		
Hyper-mutated			33.214	0.000
Yes	15	47		
No	6	219		
CIMP Category			20.582	0.000
CIMP	13	62		
EBV-CIMP	4	23		
Other	4	181		
*ARID1A* Mutation			21.618	0.000
Yes	16	73		
No	5	193		
*PIK3CA* Mutation			31.188	0.000
Yes	14	43		
No	7	223		

CIN, chromosomal instability; EBV, Epstein-Barr virus type; GS, genome stability; MSI, microsatellite instability; MSH-H, microsatellite instability-High; MSH-L, microsatellite instability-Low; MSS, microsatellite stability; CIMP, CpG island methylator phenotype.

### LinkedOmics Data

**FIGURE 2:**
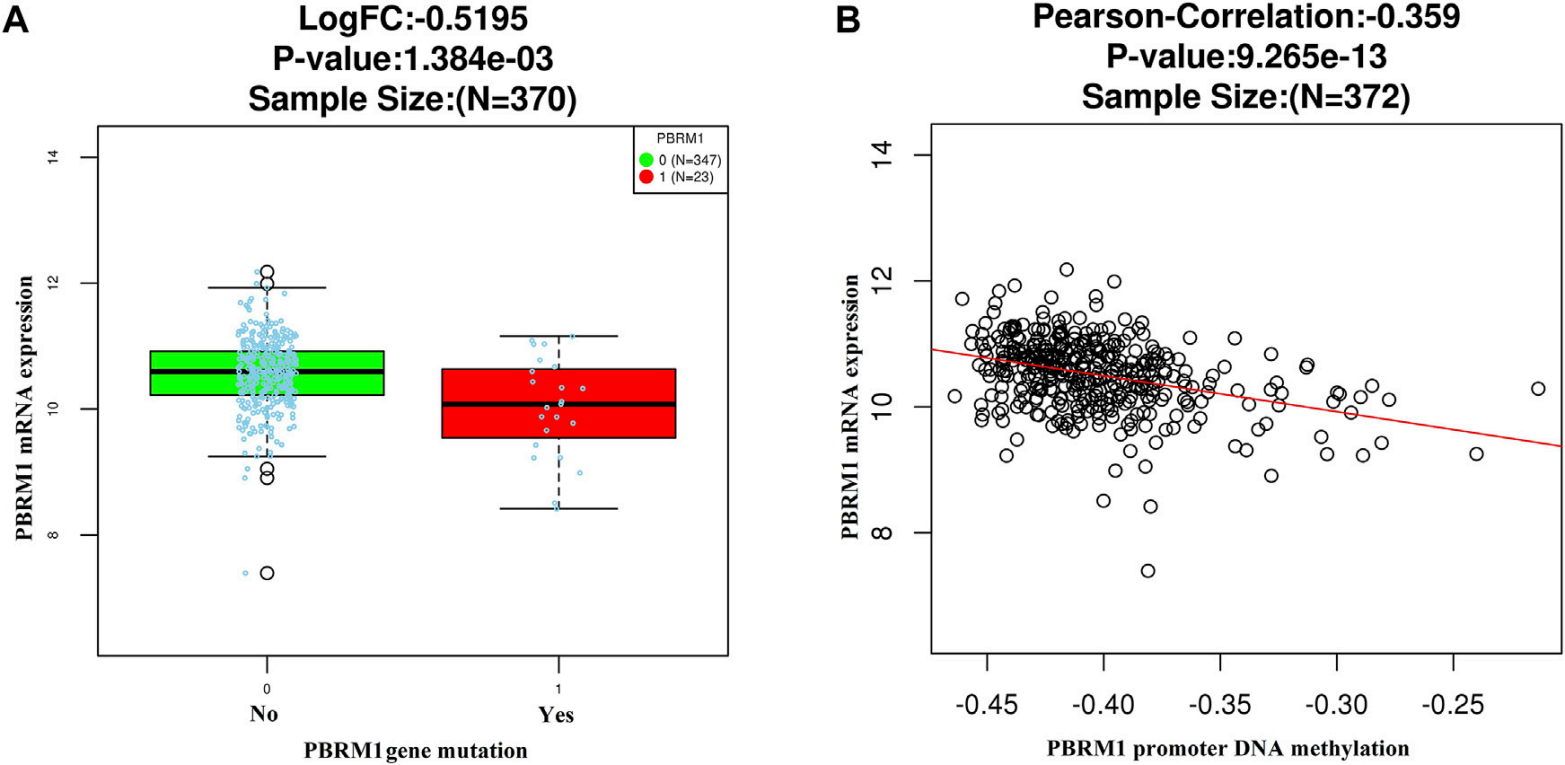
The LinkedOmics dataset shows that PBRM1 mRNA expression is significantly lower in PBRM1 mutant cases (Wilcoxon Test) **(A)** and significantly negatively correlated with PBRM1 promoter DNA methylation (Pearson Correlation test) **(B)** in GAC.

**FIGURE 3:**
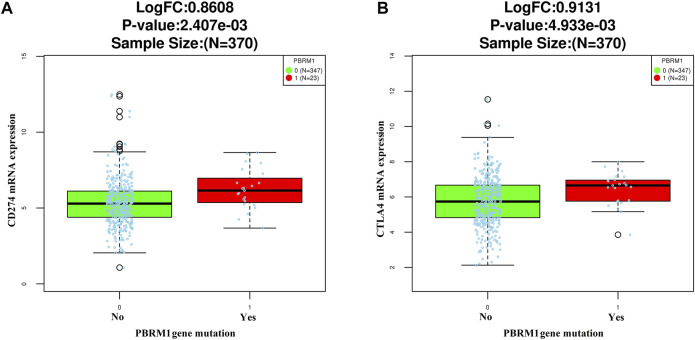
The relationship between PD-L1 (CD274) **(A)** and CTLA4 **(B)** mRNA expression and PBRM1 mutation in GAC in the LinkedOmics dataset (Wilcoxon Test).

**FIGURE 4:**
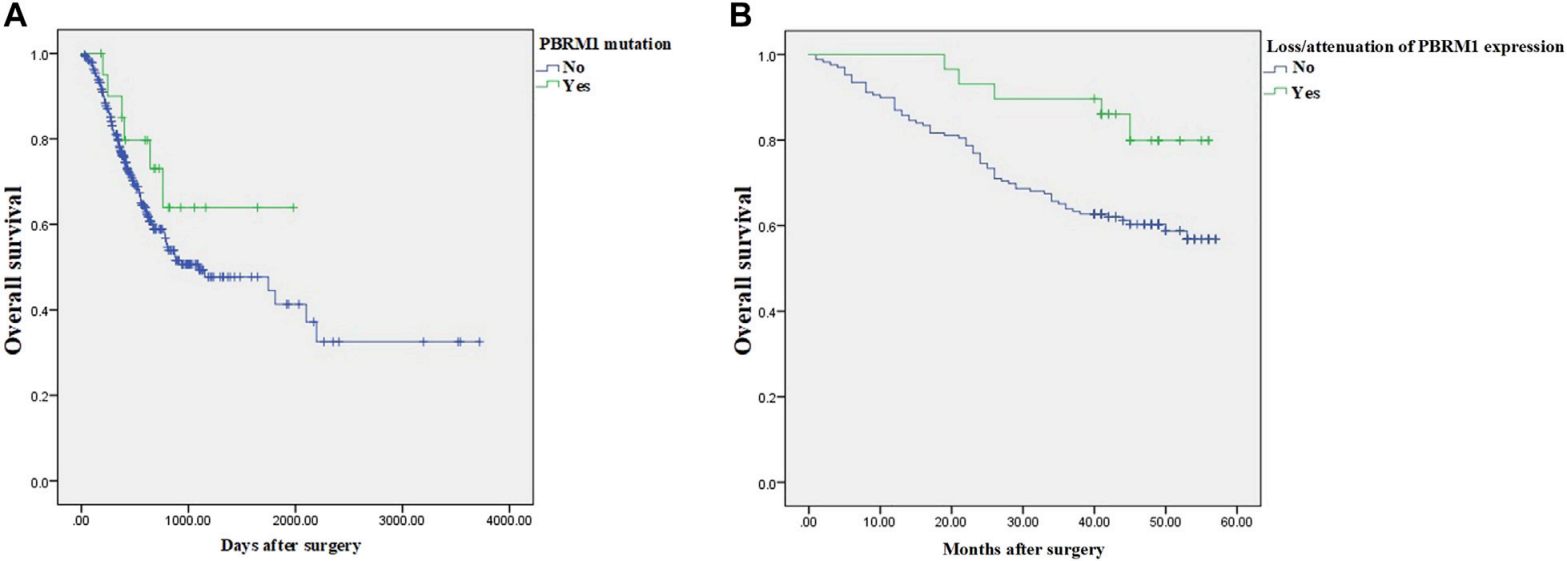
The prognosis of GAC patients with PBRM1 abnormalities. In the 354 cases of the LinkedOmics dataset, the prognosis of GAC patients with PBRM1 mutation is relatively better than that of PBRM1 wild-type patients (Cox Regression Test, *HR* = −0.5009, *p* = 0.2311) **(A)**, and the overall survival of PBRM1 expression loss/attenuation is significantly better in the 198 cases of our group (log-rank test, Chi square value = 5.159, *p* = 0.023) **(B)**.

The LinkedOmics dataset showed that PBRM1 mRNA expression was significantly lower in PBRM1 mutant cases (Figure 2A), PBRM1 promoter DNA methylation was significantly negatively correlated with PBRM1 mRNA expression (Pearson correlation = −0.359, *p* = 9.265e−13) (Figure 2B), and PBRM1 mutation cases had significantly higher mRNA expression levels of the immunosuppressive molecules PD-L1 (CD274) and CTLA4 (both *p* <0.01) (Figures 3A,B) in GAC. The prognosis of GAC patients with PBRM1 mutation was relatively better than that of PBRM1 wild-type patients (*HR* = -0.5009, *p* = 0.2311) (Figure 4A).

### TISIDB Database Analysis

**FIGURE 5:**
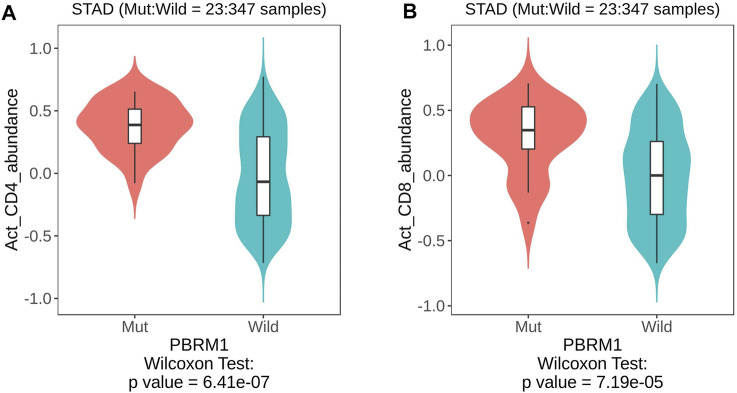
The TISIDB database shows that PBRM1 mutation is significantly associated with activated CD4 **(A)** and activated CD8 T cells **(B)** (Wilcoxon Test).

Analysis of the TISIDB database showed that PBRM1 abnormalities (including gene expression, mutation, CNA and gene methylation) were significantly associated with a variety of TILs, including activated CD4 (Figure 5A) and CD8 T cells (Figure 5B), activated dendritic cells, monocytes, dendritic cells and natural killer cells (NK cells) (Table 2), indicating that they can significantly contribute to tumor immune activity.

**TABLE 2:** The relationship between various types of PBRM1 Abnormality and TIL in GAC analyzed by the TISIDB database.

type *p* value TIL	Mutation^a^	CNA^b^	Gene methylation^b^	Gene expression^b^
Activated CD4 T cells	6.41e−07	0.13 (rho = 0.075)	0.532 (rho = −0.032)	0.00288 (rho = 0.146)
Activated CD8 T cells	7.19e−05	0.366 (rho = 0.045)	0.706 (rho = −0.02)	0.00129 (rho = −0.158)
Activated dendritic cells	0.0382	0.000749 (rho = 0.165)	0.408 (rho = −0.043)	0.222 (rho = −0.06)
Monocytes	0.0171	0.0182 (rho = 0.116)	0.000329 (rho = 0.186)	1.01e-05 (rho = −0.215)
NK cells	0.0745	0.0488 (rho = 0.099)	0.14 (rho = 0.077)	0.897 (rho = −0.006)

a Based on Wilcoxon Test. There is a positive correlation between TIL and PBRM1 mutation status.

b Based on Spearman Correlation Test. The positive or negative of the correlation coefficient rho indicated the positive or negative of the correlation.

### The Relationship Between Loss/Attenuation of PBRM1 Expression and Clinicopathology and Prognosis in Our Group

**FIGURE 6:**
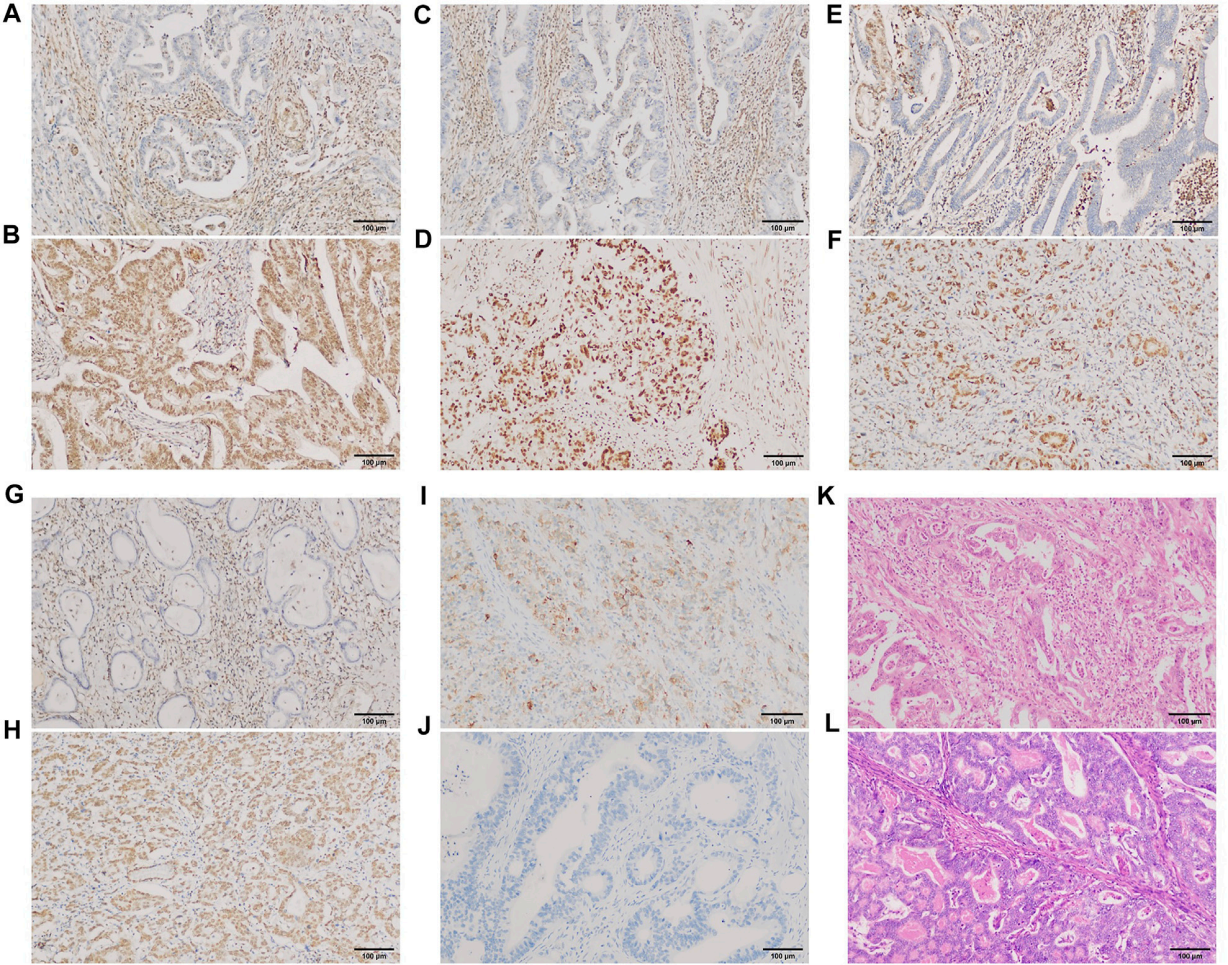
PBRM1, MMR, and PD-L1 expression and iTIL of GAC in our group. PBRM1 **(A)**, MLH1 **(C)**, MSH2 **(E)** and PMS2 **(G)** nuclear expression was lost [**(B,D,F,H)** showed that the expression of the 4 markers were retained, respectively)]; PD-L1 **(I)** cytoplasmic expression was expressed [**(J)** showed negative expression of PD-L1]. Using the EnVision method. Intra-tumoral TILs were ≥5 lymphocytes per high-power field **(K)** [**(L)** showed no iTILs] (HE staining). ×200 magnification.

Of the 198 cases in our group, PBRM1 expression was lost/attenuated in 29 (14.6%) (Figure 6A). The intestinal type with loss/attenuation of PBRM1 expression was more common, and the TNM stage was earlier (Table 3). Loss/attenuation of PBRM1 expression was significantly positively associated with dMMR (Figures 6C,E,G), PD-L1 expression (Figure 6I) and iTIL (Figure 6K) (Table 3).

**TABLE 3:** Correlation between PBRM1 expression loss/attenuation and clinicopathological features in 198 GC cases in our group.

Clinicopathological parameters	Loss/attenuation of PBRM1 expression		χ^2^ value	*p* value
	Yes	No		
Age			0.866	0.352
≤60	4	36		
>60	25	133		
Sex			0.050	0.824
Male	20	113		
Female	9	56		
Tumor site			0.340	0.844
Upper 1/3	14	72		
Middle 1/3	7	47		
Lower 1/3	8	50		
Tumor size (cm)			0.809	0.369
≤3	11	50		
>3	18	119		
Lauren type			4.961	0.084
Intestinal type	23	101		
Diffuse type	1	29		
Mixed type	5	39		
Histological differentiation			3.507	0.173
Well differentiated	2	3		
Moderately differentiated	16	82		
Poorly differentiated	11	84		
T stage			1.946	0.163
T1-2	11	43		
T3-4	18	126		
Lymph node metastasis			0.540	0.463
No	12	58		
Yes	17	111		
TNM stage			3.063	0.080
1-2	19	81		
3-4	10	88		
MMR protein expression			6.084	0.019
Intact	20	146		
Loss	9	23		
PD-L1 expression			4.081	0.043
Positive (CPS ≥ 1)	7	18		
Negative (CPS < 1)	22	151		
sTIL			2.018	0.155
Less	12	94		
More	17	75		
iTIL			5.433	0.020
No	17	133		
Yes	12	36		

As of the end of follow-up, the 3-year OS rate of 29 GAC patients with loss/attenuation of PBRM1 expression was 89.7%, while the 3-year OS rate of 169 GAC patients without PBRM1 expression loss/attenuation was 63.9%. Kaplan–Meier survival analysis showed (Figure 4B) that the overall survival of the former was significantly better than that of the latter (Chi square value = 5.159, *p* = 0.023).

In addition to loss/attenuation of PBRM1 expression, tumor size, depth of invasion, histological grade, lymph node metastasis, clinical stage, MSI, sTIL, and iTIL were statistically significant factors for survival time. The above 9 factors were included in Cox multivariate regression analysis, and the results showed that lymph node metastasis, clinical stage and iTIL were independent factors affecting the prognosis of GAC (Table 4). Loss/attenuation of PBRM1 expression did not remain an independent favorable indicator for overall survival, would be analyzed in the Discussion section.

**TABLE 4:** The variables closely related to the overall survival rate of GAC in Cox multivariate regression analysis.

Variable	*B*	SE	Wald	df	*p* value	Exp(*B*)	95%*CI*	
							Lower	Upper
Lymph node metastasis	1.317	0.646	4.161	1	0.041	3.732	1.053	13.229
Clinical stage	1.405	0.430	10.689	1	0.001	4.074	1.755	9.458
iTIL	−0.836	0.400	4.363	1	0.037	0.433	0.198	0.950

### Sequencing Results of 7 Gastric Adenocarcinoma Cases of PBRM1 Expression Loss in Our Group

Among the 7 GAC cases with loss of PBRM1 expression in our group, 3 cases showed PBRM1 mutations, including missense mutations and splicing mutations, and 2 cases had chromosome 3p21.1 deletions (the locus of the PBRM1 gene). Mutations in other SWI/SNF chromatin remodeling subunits are often detected simultaneously in the same tumor (Table 5), including ARID2, PHF10, SMARCC2, SMARCA2, SMARCC1, SMARCD3, SMARCE1, ARID1A, ARID1B, DPF1, and GLTSCR1, involving missense mutation, in frame del, splice region, nonsense mutation, frame shift del, frame shift ins, in frame ins and splice site, the former being more common.

**TABLE 5:** Mutation of SWI/SNF subunit genes in 7 GC cases with loss of PBRM1 expression in our group.

Case no.	Gene	HGVS	Variant classification
1	*PBRM1*	c.996-5dupT	Splice region
	*SMARCA2*	c.3283C > T (p.R1095C)	Missense nutation
	*SMARCC2*	c.3274_3276delCCT (p.P1092del)	In frame del
	*SMARCD3*	c.291-6G > A	Splice region
	*ARID1A*	c.3999_4001delGCA (p.Q1334del)	In frame del
	*ARID1B*	c.384_386delGCA (p.Q131del)	In frame del
	*DPF1*	c.31G > C (p.G11R)	Missense mutation
	*GLTSCR1*	c.2761A > C (p.T921P)	Missense mutation
2	*PBRM1*	c.996-4G > T	Splice region
	*PBRM1*	c.3971G > C (p.G1324A)	Missense mutation
	*SMARCC1*	c.2239G > A (p.A747T)	Missense mutation
	*ARID1A*	c.3999_4001delGCA (p.Q1334del)	In frame del
	*ARID1B*	c.285_287delCCA(p.H96del)	In frame del
	*GLTSCR1*	c.3832G > A (p.A1278T)	Missense mutation
3	*PBRM1*	c.142G > T (p.A48S)	Missense mutation
	*SMARCA2*	c.2416-7C > T	Splice region
	*SMARCA2*	c.4347C > A (p.F1449L)	Missense mutation
	*SMARCA2*	c.3274_3276delCCT (p.P1092del)	In frame del
	*SMARCC2*	c.3274_3276delCCT (p.P1092del)	In frame del
	*PHF10*	c.544-4C > T	Splice region
	*ARID1A*	c.1489delC (p.Q497Nfs*122)	Frame shift del
	*ARID1A*	c.3999_4001delGCA (p.Q1334del)	In frame del
	*ARID1A*	c.1489C > G (p.Q497E)	Missense mutation
	*GLTSCR1*	c.2594A > C (p.Q865P)	Missense mutation
	*GLTSCR1*	c.2761A > C (p.T921P)	Missense mutation
4	*SMARCC2*	c.3422A > C (p.N1141T)	Missense mutation
	*SMARCC2*	c.932C > A (p.A311E)	Missense mutation
	*ARID1B*	c.2081G > A (p.S694N)	Missense mutation
	*ARID1B*	c.4855+1G > A (p.X1619_splice)	Splice site
	*GLTSCR1*	c.2594A > C (p.Q865P)	Missense mutation
	*GLTSCR1*	c.2761A > C (p.T921P)	Missense mutation
	*GLTSCR1*	c.2777T > C (p.L926P)	Missense mutation
5	*PHF10*	c.544-5dupT	Splice region
	*SMARCC1*	c.3248C > T (p.P1083L)	Missense mutation
	*ARID1B*	c.384_386dupGCA (p.Q131dup)	In frame ins
	*BRD9*	c.178C > T (p.R60*)	Nonsense mutation
6	*ARID2*	c.3203G > A (p.R1068H)	Missense mutation
	*SMARCC2*	c.3274_3276delCCT (p.P1092del)	In frame del
	*SMARCE1*	c.988G > A (p.E330K)	Missense mutation
	*ARID1A*	c.3999_4001delGCA (p.Q1334del)	In frame del
	*ARID1A*	c.5554_5555insTTGAG (p.T1852Ifs*33)	Frame shift ins
7	*GLTSCR1*	c.3115G > A (p.A1039T)	Missense mutation

**FIGURE 7:**
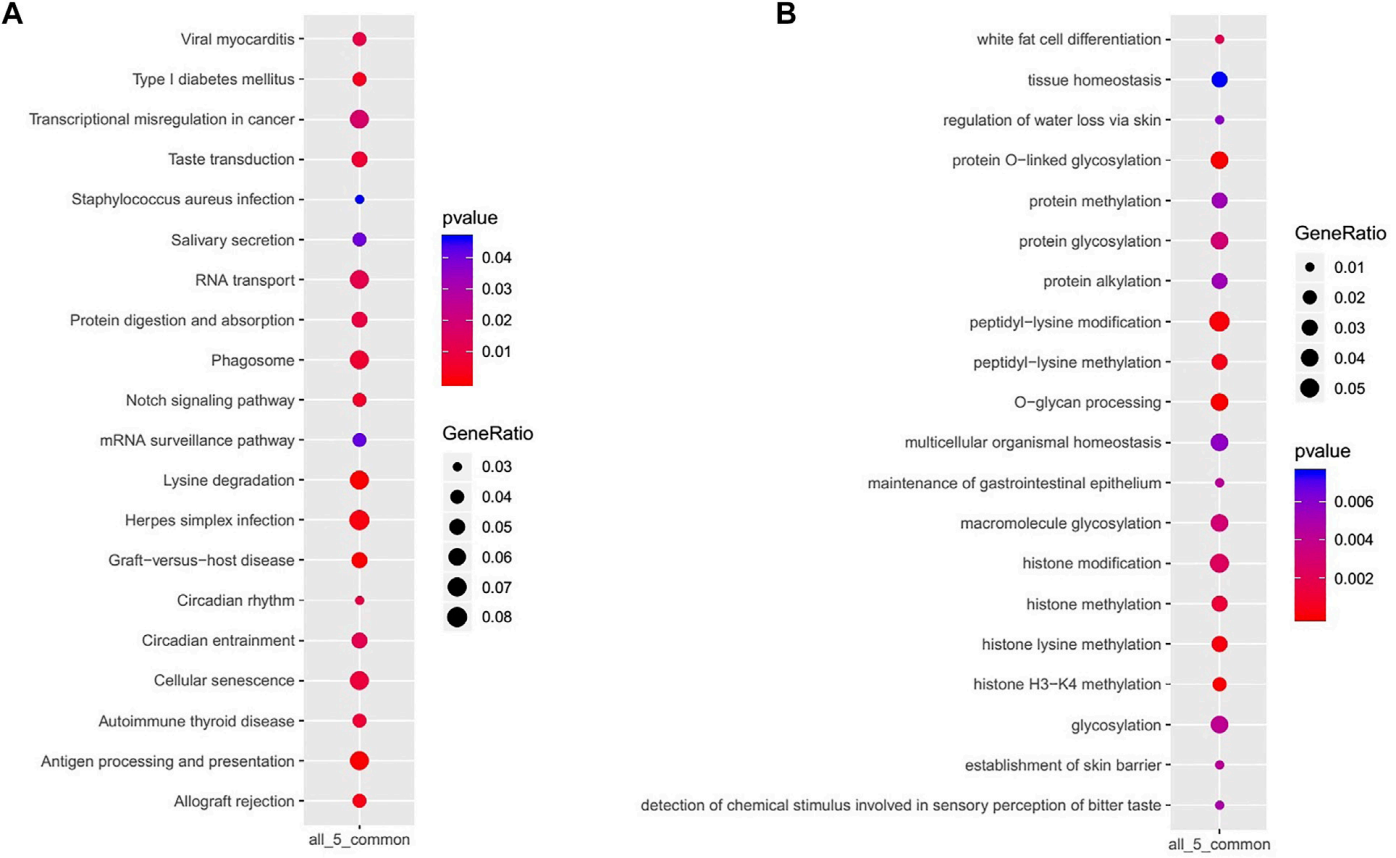
KEGG signaling pathways **(A)** and biological processes **(B)** mainly enriched in GACs with PBRM1 expression loss.

KEGG signaling pathways enriched in GACs with PBRM1 expression loss mainly included lysine degradation, antigen processing and presentation, the Notch signaling pathway and phagosome-related genes (Figure 7A), and the biological processes mainly included O-glycan processing, histone H3-K4 methylation, peptidyl-lysine modification, histone lysine methylation and methylation, and protein and macromolecule glycosylation (Figure 7B).

## Discussion

Analysis of public databases and the data of GAC cases collected in our group showed that the PBRM1 gene was significantly abnormal in a small number of GACs (approximately 10%), including gene deletion/mutation, methylation and loss/attenuation of expression. According to the literature, PBRM1 inactivating mutation or expression loss exists in approximately 40% of clear cell renal carcinoma, 32% of cholangiocarcinoma and 83% of epithelioid sarcoma (12), which is an important tumor driver gene, suggesting that it may be an important driving carcinogenesis factor for a small portion of GAC. This study also showed that PBRM1 deletion/mutation or loss/attenuation of PBRM1 expression often coexists with other SWI/SNF subunit mutations, indicating that the functions of SWI/SNF subunits may have mutual compensatory effects, improving the dysfunction of the complex caused by the inactivation of a certain subunit function, and its dysfunction may be associated with simultaneous abnormalities of multiple subunits (13). At the same time, according to our sequencing and LinkedOmics data results, we also found that the loss or downregulation of PBRM1 expression may involve multiple mechanisms, including deletion/mutation and CpG island methylation in the gene promoter region.

Interestingly, prognostic analysis showed that abnormality of the PBRM1 gene was a favorable prognostic factor, and multivariate regression analysis showed that iTILs, which were closely positively related to PBRM1 expression loss/attenuation, were an independent prognostic factor. Comprehensive public databases and the data in our group found that the abnormal SWI/SNF subunit PBRM1 was closely related to tumor immune microenvironment-related factors, such as high expression of the immunosuppressive molecules PD-L1 and CTLA4, high sTIL, and iTIL. This indicates that there may be a balance between carcinogenesis and tumor suppressor immune activity of PBRM1 abnormalities (14), thereby affecting the overall biological behavior and prognosis of tumors. A study using a genome-wide CRISPR–Cas9 screen found that PBRM1 expression was inversely correlated with the expression of T-cell cytotoxicity genes in a variety of cancers. Low expression of PBRM1 was associated with higher cytotoxic activity and degree of infiltration of CD8 T cells. Loss of PBAF function increases tumor cell sensitivity to interferon-gamma, resulting in increased secretion of chemokines that recruit effector T cells, an increased percentage of dendritic cells and a higher ratio of tumor-inhibitory M1-like macrophages to tumor-promoting M2-like macrophages. Single-cell RNA-seq analysis of sorted CD45^+^ immune cells showed that gene expression signatures were associated with tumor immunity (IFN-γ response, IFN-α response, and tumor necrosis factor-α signaling through NF-κB) in myeloid cells (dendritic cells and M1-like macrophages) and lymphocytes (T cells and natural killer cells) in PBRM1-deficient individuals (15). Rhabdoid tumors (RTs) are malignant tumors driven by inactivation of the SWI/SNF subunit SMARCB1. A study found that RTs in the brain with low PBAF expression had a better overall prognosis. PBRM1 knockdown resulted in decreased immunosuppressive cytokines, and PBRM1 levels in tumor tissues were negatively correlated with CD8 cytotoxic T-cell (CTC) infiltration (16).

Studies have shown that the mRNA and protein expression levels of the immune checkpoint molecules PD-L1 and CTLA4 are also closely related to high TILs, an active immune response, and a “hot” immune microenvironment, which is beneficial for the tumor-targeted immune response or immunotherapy (17, 18). iTILs can further enhance antitumor activity through direct contact with cancer cells. These include conventional CD4^+^ or CD8^+^ T cells (also known as inducible iTILs) as well as unconventional γδ T cells or CD4^−^CD8αβ-TCRαβ+ T cells (double negative T) (also known as native iTILs). Native iTILs possess autoreactive T-cell receptors (TCRs), granzymes A and B, and high cytotoxic activity (19). We preliminarily explored the specific types of TILs associated with abnormal PBRM1 genes in GAC through the TISIDB database, including activated CD4 and CD8 T cells, activated dendritic cells, monocytes, and NK cells. Therefore, an abnormal PBRM1 gene can lead to a more favorable tumor microenvironment and make tumor cells more sensitive to immune cell-mediated cytotoxicity, such as that driven by T cells.

There is a significant correlation between abnormal SWI/SNF subunit and MSI-H, and both are related to PD-L1 expression, high tumor genome mutation rate, and high TIL, resulting in high tumor immune activity. There are several hypotheses regarding the causal affiliation of the two: 1) SWI/SNF complex subunit gene mutations may be caused by MSI; 2) the two may be a reflection of genome-wide hypermethylation, that is, a CpG island methylation phenotype; and 3) SWI/SNF complex deficiency leads to impaired MMR, mutation or MLH1 promoter methylation and epigenetic alterations (20, 21). Studies have found that SWI/SNF subunit ARID1A deficiency attenuates mismatch repair (MMR) capacity, impairs mismatch repair in a variety of cancers, leads to genomic alterations in microsatellite instability (22, 23), increases TMB, increases neoantigen presentation, increases TILs, and makes tumors susceptible to immune checkpoint blockade. In low MSI/microsatellite stable subtype gastrointestinal cancer (excluding the influence of MSI-H), ARID1A mutant cases still had significantly higher tumor immune activity scores than ARID1A wild type tumors, including high TMB, PD-L1 expression and CTC infiltration (24, 25). Therefore, the effects of abnormal SWI/SNF subunits (including the PBRM1 gene) and MSI on tumor immune activity may be both synergistic and independent, and the former may be another important factor leading to increased tumor immunity in addition to MSI-H. In pancreatic cancer with SWI/SNF subunit loss, some cases that were not accompanied by MSI-H, high TMB and/or PD-L1 expression also responded to immune checkpoint inhibitors (26). Therefore, abnormal SWI/SNF subunits, including PBRM1, should be considered as other important markers in addition to MSI and PD-L1 expression in GAC immunotherapy.

In clinical practice, it is difficult to recover from the abnormal deletion of gene products, but abnormal SWI/SNF subunits often co-occur with abnormalities of other molecular signaling pathways. The use of specific synthetic lethal relationships is an effective approach to its treatment and has now become a hot field of precision therapy (27). This study showed that PBRM1 deletion/mutation is closely related to PIK3CA mutation and that KEGG signaling pathways enriched for PBRM1 expression loss include lysine degradation, antigen processing and presentation, the Notch signaling pathway and phagosome-related genes in GACs. In the future, it may be applied in the treatment of synthetic lethal mechanisms or as a predictive marker for anti-gene targeted therapy in GAC.

In conclusion, PBRM1 gene abnormalities may play an important carcinogenic role in some gastric cancer subgroups and may affect their tumor immune activity, thereby influencing the clinicopathological and overall prognosis of GAC. The detection of PBRM1 gene abnormalities may be an effective predictive marker in immunotherapy, and harnessing the synthetic lethal relationship associated with PBRM1 gene abnormalities may also be a potential novel therapeutic strategy in GAC.

## Data Availability

The original contributions presented in the study are included in the article/Supplementary Material, further inquiries can be directed to the corresponding author.

## References

[1] Joshi SS Badgwell BD. Current Treatment and Recent Progress in Gastric Cancer. CA Cancer J Clin (2021) 71(3):264–79. 10.3322/caac.21657 33592120 PMC9927927

[2] Sasaki M Ogiwara H. Synthetic Lethal Therapy Based on Targeting the Vulnerability of SWI/SNF Chromatin Remodeling Complex‐deficient Cancers. Cancer Sci (2020) 111(3):774–82. 10.1111/cas.14311 31955490 PMC7060479

[3] Liu X-D Kong W Peterson CB McGrail DJ Hoang A Zhang X PBRM1 Loss Defines a Nonimmunogenic Tumor Phenotype Associated with Checkpoint Inhibitor Resistance in Renal Carcinoma. Nat Commun (2020) 11(1):2135. 10.1038/s41467-020-15959-6 32358509 PMC7195420

[4] Gao J Aksoy BA Dogrusoz U Dresdner G Gross B Sumer SO Integrative Analysis of Complex Cancer Genomics and Clinical Profiles Using the cBioPortal. Sci Signal (2013) 6(269):pl1. 10.1126/scisignal.2004088 23550210 PMC4160307

[5] Vasaikar SV Straub P Wang J Zhang B. LinkedOmics: Analyzing Multi-Omics Data within and across 32 Cancer Types. Nucleic Acids Res (2018) 46(D1):D956–D963. 10.1093/nar/gkx1090 29136207 PMC5753188

[6] Ru B Wong CN Tong Y Zhong JY Zhong SSW Wu WC TISIDB: An Integrated Repository portal for Tumor-Immune System Interactions. Bioinformatics (2019) 35(20):4200–2. 10.1093/bioinformatics/btz210 30903160

[7] Huang S-C Ng K-F Chang IY-F Chang C-J Chao Y-C Chang S-C The Clinicopathological Significance of SWI/SNF Alterations in Gastric Cancer Is Associated with the Molecular Subtypes. PLoS One (2021) 16(1):e0245356. 10.1371/journal.pone.0245356 33481850 PMC7822341

[8] Hendry S Salgado R Gevaert T Russell PA John T Thapa B Assessing Tumor-Infiltrating Lymphocytes in Solid Tumors: A Practical Review for Pathologists and Proposal for a Standardized Method from the International Immuno-Oncology Biomarkers Working Group: Part 2: TILs in Melanoma, Gastrointestinal Tract Carcinomas, Non-small Cell Lung Carcinoma and Mesothelioma, Endometrial and Ovarian Carcinomas, Squamous Cell Carcinoma of the Head and Neck, Genitourinary Carcinomas, and Primary Brain Tumors. Adv Anat Pathol (2017) 24(6):311–35. 10.1097/PAP.0000000000000161 28777143 PMC5638696

[9] González IA Bauer PS Liu J Chatterjee D. Intraepithelial Tumour Infiltrating Lymphocytes Are Associated with Absence of Tumour Budding and Immature/myxoid Desmoplastic Reaction, and with Better Recurrence‐free Survival in Stages I-III Colorectal Cancer. Histopathology (2021) 78(2):252–64. 10.1111/his.14211 32654226 PMC7775349

[10] Jun S-Y Park ES Lee JJ Chang H-K Jung ES Oh Y-H Prognostic Significance of Stromal and Intraepithelial Tumor-Infiltrating Lymphocytes in Small Intestinal Adenocarcinoma. Am J Clin Pathol (2020) 153(1):105–18. 10.1093/ajcp/aqz136 31576398

[11] Xu H Zhu X Bao H Wh Shek T Huang Z Wang Y Genetic and Clonal Dissection of Osteosarcoma Progression and Lung Metastasis. Int J Cancer (2018) 143(5):1134–42. 10.1002/ijc.31389 29569716

[12] Savas S Skardasi G. The SWI/SNF Complex Subunit Genes: Their Functions, Variations, and Links to Risk and Survival Outcomes in Human Cancers. Crit Rev Oncol Hematol (2018) 123:114–31. 10.1016/j.critrevonc.2018.01.009 29482773

[13] Pierre RS Kadoch C. Mammalian SWI/SNF Complexes in Cancer: Emerging Therapeutic Opportunities. Curr Opin Genet Dev (2017) 42:56–67. 10.1016/j.gde.2017.02.004 28391084 PMC5777332

[14] Hu B Lin JZ Yang XB Sang XT. The Roles of Mutated SWI/SNF Complexes in the Initiation and Development of Hepatocellular Carcinoma and its Regulatory Effect on the Immune System: A Review. Cell Prolif (2020) 53(4):e12791. 10.1111/cpr.12791 32162380 PMC7162795

[15] Pan D Kobayashi A Jiang P Ferrari de Andrade L Tay RE Luoma AM A Major Chromatin Regulator Determines Resistance of Tumor Cells to T Cell-Mediated Killing. Science (2018) 359(6377):770–5. 10.1126/science.aao1710 29301958 PMC5953516

[16] Panwalkar P Pratt D Chung C Dang D Le P Martinez D SWI/SNF Complex Heterogeneity Is Related to Polyphenotypic Differentiation, Prognosis, and Immune Response in Rhabdoid Tumors. Neuro Oncol (2020) 22(6):785–96. 10.1093/neuonc/noaa004 31912158 PMC7283032

[17] Xing X Guo J Ding G Li B Dong B Feng Q Analysis of PD1, PDL1, PDL2 Expression and T Cells Infiltration in 1014 Gastric Cancer Patients. Oncoimmunology (2017) 7(3):e1356144. 10.1080/2162402X.2017.1356144 29399387 PMC5790386

[18] Kim H Heo YJ Cho YA Kang SY Ahn S Kim K-M. Tumor Immune Microenvironment Is Influenced by Frameshift Mutations and Tumor Mutational burden in Gastric Cancer. Clin Transl Oncol (2022) 24(3):556–67. 10.1007/s12094-021-02714-6 34767183

[19] Morikawa R Nemoto Y Yonemoto Y Tanaka S Takei Y Oshima S Intraepithelial Lymphocytes Suppress Intestinal Tumor Growth by Cell-To-Cell Contact via CD103/E-Cadherin Signal. Cell Mol Gastroenterol Hepatol (2021) 11(5):1483–503. 10.1016/j.jcmgh.2021.01.014 33515805 PMC8025200

[20] Schallenberg S Bork J Essakly A Alakus H Buettner R Hillmer AM Loss of the SWI/SNF-ATPase Subunit Members SMARCF1 (ARID1A), SMARCA2 (BRM), SMARCA4 (BRG1) and SMARCB1 (INI1) in Oesophageal Adenocarcinoma. BMC Cancer (2020) 20(1):12. 10.1186/s12885-019-6425-3 31906887 PMC6945480

[21] Peng L Li J Wu J Xu B Wang Z Giamas G A Pan-Cancer Analysis of SMARCA4 Alterations in Human Cancers. Front Immunol (2021) 12:762598. 10.3389/fimmu.2021.762598 34675941 PMC8524462

[22] Wang L Yang L Wang C Zhao W Ju Z Zhang W Inhibition of the ATM/Chk2 axis Promotes cGAS/STING Signaling in ARID1A-Deficient Tumors. J Clin Invest (2020) 130(11):5951–66. 10.1172/JCI130445 33016929 PMC7598069

[23] Shen J Ju Z Zhao W Wang L Peng Y Ge Z ARID1A Deficiency Promotes Mutability and Potentiates Therapeutic Antitumor Immunity Unleashed by Immune Checkpoint Blockade. Nat Med (2018) 24(5):556–62. 10.1038/s41591-018-0012-z 29736026 PMC6076433

[24] Tokunaga R Xiu J Goldberg RM Philip PA Seeber A Battaglin F The Impact of ARID1A Mutation on Molecular Characteristics in Colorectal Cancer. Eur J Cancer (2020) 140:119–29. 10.1016/j.ejca.2020.09.006 33080474 PMC8009046

[25] Li L Li M Jiang Z Wang X. *ARID1A* Mutations are Associated with Increased Immune Activity in Gastrointestinal Cancer. Cells (2019) 8(7):678. 10.3390/cells8070678 PMC6678467 31277418

[26] Botta GP Kato S Patel H Fanta P Lee S Okamura R SWI/SNF Complex Alterations as a Biomarker of Immunotherapy Efficacy in Pancreatic Cancer. JCI Insight (2021) 6(18):e150453. 10.1172/jci.insight.150453 34375311 PMC8492298

[27] Xu S Tang C. The Role of *ARID1A* in Tumors: Tumor Initiation or Tumor Suppression? Front Oncol (2021) 11:745187. 10.3389/fonc.2021.745187 34671561 PMC8521028

